# How do socioeconomic inequalities and preterm birth interact to modify health and education outcomes? A narrative systematic review

**DOI:** 10.1136/bmjopen-2024-084147

**Published:** 2025-01-25

**Authors:** Philip McHale, Katie Fahy, Andy Pennington, Daniela K Schlüter, Ben Barr, David Taylor-Robinson

**Affiliations:** 1 Department of Public Health, Policy and Systems, University of Liverpool, Liverpool, UK

**Keywords:** PUBLIC HEALTH, Systematic Review, PAEDIATRICS

## Abstract

**Abstract:**

**Objectives:**

How are socioeconomic inequalities modified by, or how do they interact with, preterm birth?

**Design:**

Narrative systematic review of quantitative observational studies of an interaction, or effect modification, between preterm birth and socioeconomic status.

**Data sources:**

Five databases were searched for studies published between January 2000 and June 2020. Title and abstract were reviewed to identify articles for dual screening. All included studies were citation searched.

**Eligibility criteria:**

Inclusion criteria were comparison across socioeconomic status and gestational age, interaction between the two, or stratification by either, and health or education as outcome.

**Data extraction and synthesis:**

Data extracted included study design, sample size, outcome, interaction measure, effect and significance. Included studies were assessed for methodological quality and synthesised narratively.

**Results:**

After searches, 52 studies were identified for full-text screening and, with supplementary citation searches, we identified 21 included studies. Eighteen studies studied interaction between gestational age or preterm birth, and socioeconomic status. Three groups of outcomes were identified: cognitive, mental health and developmental. Age at outcome measurement was divided into four categories: preschool, primary school (5–11), secondary school (11–18) and post school (18–29). Seven studies found a significant interaction between the effect of preterm birth and socioeconomic status. Six of these interactions demonstrate that the negative influence of low socioeconomic status was stronger for those born preterm (and vice versa) for cognitive and mental health outcomes, all in studies with a sample size of more than 100 000. One study found that negative effects of low socioeconomic status were reduced for those born preterm (and vice versa) for communication delay.

**Conclusions:**

Our findings suggest that the impact of low socioeconomic status on cognitive and mental health outcomes is exacerbated by preterm birth. The remaining evidence suggests the effects are not modified; however, this is potentially due to underpowered studies. Public health action is indicated to support babies born preterm, particularly for disadvantaged families, to improve educational attainment and mental health.

**PROSPERO registration number:**

CRD42020203613.

**STRENGTHS AND LIMITATIONS OF THIS STUDY**

- Our study used extensive searches of multiple databases and supplementary searches, so we are confident we have identified the most relevant studies.
- Our methodological quality appraisal included scoring of interaction analysis, increasing the relevance of the quality scores to our study.
- We limited screening to titles and abstracts; therefore, it is possible we may have excluded some papers that mentioned interaction or stratification elsewhere, as an additional element of analysis.
- Reporting of interaction was frequently lacking important information to support assessment.

## Introduction

 Preterm birth (PTB), or low gestational age (GA) at birth, refers to births at GA before 37 weeks and is a significant public health problem. This affects more than one in 10 births globally and approximately seven to eight per cent of births in the UK.^1^ Significant socioeconomic inequalities in PTB are consistently demonstrated, with women in low socioeconomic status (SES) delivering preterm at significantly higher rates.^2 3^ Low SES is a major driver of PTB risk.^4^

Being born preterm is a significant risk for health conditions, worse developmental and educational outcomes, and a leading cause of childhood mortality.^5 6^ Notably, the negative effects of PTB on cognitive outcomes remain after controlling for underlying disability, which suggests that PTB has a direct effect on this outcome.^7^ Additionally, low SES is associated with worse educational outcomes in children born preterm.^8^

These negative consequences of PTB are socially patterned; children growing up in low socioeconomic circumstances have worse educational and health outcomes.^9 10^ Given low maternal SES is associated with increased risk of PTB, there is potentially a significant cohort of children who are doubly exposed to both these risks, increasing the risks for the negative outcomes.

There is evidence that the effects of perinatal factors (exposure) on health and educational outcomes are modified by other factors, so-called interaction. Age at school entry modifies the effect of PTB on educational attainment,^11^ sex modifies the effect of being born small for GA on risk of asthma^12^ and the effect of PTB on neurodevelopmental outcomes,^13^ and parental education modifies the effect of birth weight on educational attainment.^14^

It is therefore important to identify whether, and how, this double exposure of low SES and PTB leads to a modification of the risks. Interaction is defined based on the scale it is measured on:

- Additive: presence of interaction on the absolute scale—the probability of outcome when doubly exposed (low SES and PTB) minus the probabilities when both exposures occur independently. If greater than 0, then superadditive (effects of both exposures together greater than the sum of each independently). If less than 0, then subadditive (effects of both exposures together less than the sum of each independently).
- Multiplicative: presence of interaction on the relative scale—the relative measure of outcome when doubly exposed (low SES and PTB) divided by the relative measures when both exposures occur independently, all compared with when neither exposure occurs. If greater than 1, then supermultiplicative (ie, effects exacerbated); if less than 1, then submultiplicative (effects of both exposures together less than the sum of each independently, that is, effects mitigated).^15^

To compare both interaction and effect modification, we consider effect modification as either the effect of PTB across different SES strata, or vice versa.^16^

Understanding the nature of this interaction is key to informing follow-up and support for children born preterm. For example, the presence of PTB may exacerbate the consequences of low SES on later life outcomes, and vice versa, leading to greater inequalities, and indicating specific support needs for children who are exposed to both. The theoretical basis for this research is the Diderichsen model of health inequalities.^17^ This suggests that health inequalities are generated by a set of differentials, with this study focussing on the concepts of differential susceptibility (does PTB make children born in low socioeconomic circumstances more susceptible to the negative outcomes) and differential disease consequence (are the negative consequences of PTB exacerbated by being born in low socioeconomic circumstances). If an interaction between PTB and low SES exists, combined with the increased risk of PTB in low SES groups, this would perpetuate the negative outcomes and potentially exacerbate inequalities.

The current guidance for paediatric care of preterm babies focuses on surveillance and support, with limited consideration of SES.^18^ If there is a significant interaction between SES and PTB, there will be a need to consider how equitable access to healthcare and support is provided for children born preterm. The aim of this review is to identify, and synthesise, evidence for interaction between SES and PTB on health, developmental and educational outcomes.

## Methods

This review was conducted in conjunction with another review, following a similar methodology.^4^ This review sought relevant empirical studies published between January 2000 and July 2020 that address the research question: ‘How does preterm birth influence the relationship between maternal socioeconomic status and outcomes for health and education?’ The protocol was registered with the Prospective Register for Systematic Reviews (PROSPERO) (registration code: PROSPERO 2020 CRD42020203613) and can be accessed from https://www.crd.york.ac.uk/prospero/display_record.php?ID=CRD42020203613. Reporting follows Preferred Reporting Items for Systematic Reviews and Meta-Analyses (PRISMA) guidance (as per PRISMA statement, online supplemental appendix A). Deviations from the protocol (online supplemental appendix B) have not influenced our findings.

### Search strategy

Five databases were searched: Scopus, Medline, ‘Medline In Process & Other Non-Indexed Citation’, PsycInfo and Social Science Citation Index (via Web of Science). An existing review of interaction was assessed to inform search terms however no specific terms for interaction or effect modification were included in this review.^19^ Search terms followed the PICO structure (table 1) and were supplemented through Advanced Google searching. Updated rapid searches were conducted in Scopus and Advanced Google between July 2020 and July 2023, with new studies included in a separate section of the results. Search terms covered interaction or effect modification, SES and PTB (online supplemental appendix C).

**Table 1:** Inclusion/exclusion criteria for systematic review question: ‘How does preterm birth influence the relationship between maternal socioeconomic status and adolescent outcomes for health and education?’

	Include	Exclude
Population/settings	All births	–
Intervention/exposure	Preterm birth and gestational age	Other birth outcomes (eg, low birth weight)
Comparison	Comparison across socioeconomic strata	No socioeconomic comparisonOnly preterm, no comparison with full term
Outcomes	Health and educational/employment outcomes through to young adulthood	–
Publication characteristics inclusion/exclusion criteria		
Publication types	Primary studies from peer-reviewed literature, including those from reviews.Relevant secondary analyses (meta-analysis)Papers published or in-press.Working papers.	Any work that is not a primary research study, including letters, editorials, commentaries, conference proceedings, books and book chapters, meeting abstracts, lectures and addresses.Previous reviews are not eligible, but relevant reviews will be used to identify relevant primary studies.
Types of study	All study designsAnalytical techniques that are relevant: Effect modification Interaction Differential susceptibility	Other methodologiesEffect modification or interaction not specifically calculated within analysis
Year of publication	2000–2020	–
Language	English language	–

To identify further studies of interest, all included studies were hand-searched for backward citation (using reference lists) and forward citation (using Web of Science). Studies included in two systematic reviews identified were also assessed.^20 21^ Screening used EPPI-Reviewer 4 systematic review management software.^22^

### Selection

Titles and abstracts were screened and those mentioning interaction or effect modification between SES and PTB were then reviewed against the inclusion/exclusion criteria (table 1). Approximately 15% of titles and abstracts were independently screened by two reviewers, before a screening ‘calibration’ exercise. The remaining titles and abstracts were then screened by a single reviewer. The resulting papers underwent full-text screening independently by two reviewers. Any screening disagreements were resolved through discussion or recourse to a third reviewer.

### Data extraction

All data were extracted by one reviewer, then checked for accuracy and completeness independently by the other reviewer. Data extracted included study design, population, time period, sample size (large sample required to detected an interaction effect),^23^ measure of SES, measure of GA or PTB, outcome, interaction measure (additive or multiplicative), the main effects of SES and PTB, or GA, on outcome, and interaction effect and significance (the standardised metric used in synthesis, measured either by a coefficient or a qualitative description).^24^ PTB was assessed as lower GA to allow comparative synthesis between the two measures of exposure. For studies which included multiple models, we used results from the model which included both PTB/GA, the measure of SES and the interaction term for main effects, if this was available.

### Methodological quality scoring

Studies were assessed for methodological quality independently by both reviewers using a hybrid approach. This assessed the quality of the study through the risk of bias and the reporting of the interaction results. Risk of bias associated with study design was assessed using the Liverpool University Quality Assessment Tool relevant to cohort studies (online supplemental appendix D).^25^ Maximum score was 12.

### Synthesis

Studies were synthesised narratively, and results were grouped according to outcome: cognitive, developmental or mental health.^26^ They were measured at four age groups: preschool (less than 5 years old), primary school (5–11), secondary school (12–18) and post school (over 18). The prioritisation of results reporting was based on frequency of the outcome in the included studies and the quality scoring.^26 27^ Criteria for publication bias could not be calculated and meta-analysis was not feasible.^24^ Results were described as the effects of lower SES and lower GA, with higher SES/GA as the reference level (studies describing results the other way were harmonised/reversed). A harvest plot was used to display the presence or absence of a significant interaction for the three outcomes, by age, country, quality and sample size.^28^ An albatross plot could not be produced due to lack of specific information on p value size or direction of the interaction effect.^24 29^

### Patient and public involvement

Our study is part of a programme of work informed by discussions around the UNICEF Child Friendly City programme in Liverpool. Children and families encouraged us to undertake analyses focused on social inequalities impacting children’s health, including early years influences. The results of our work are feeding into ongoing discussion with young people and service providers about addressing health inequalities.

## Results

### Searches

**Figure 1:**
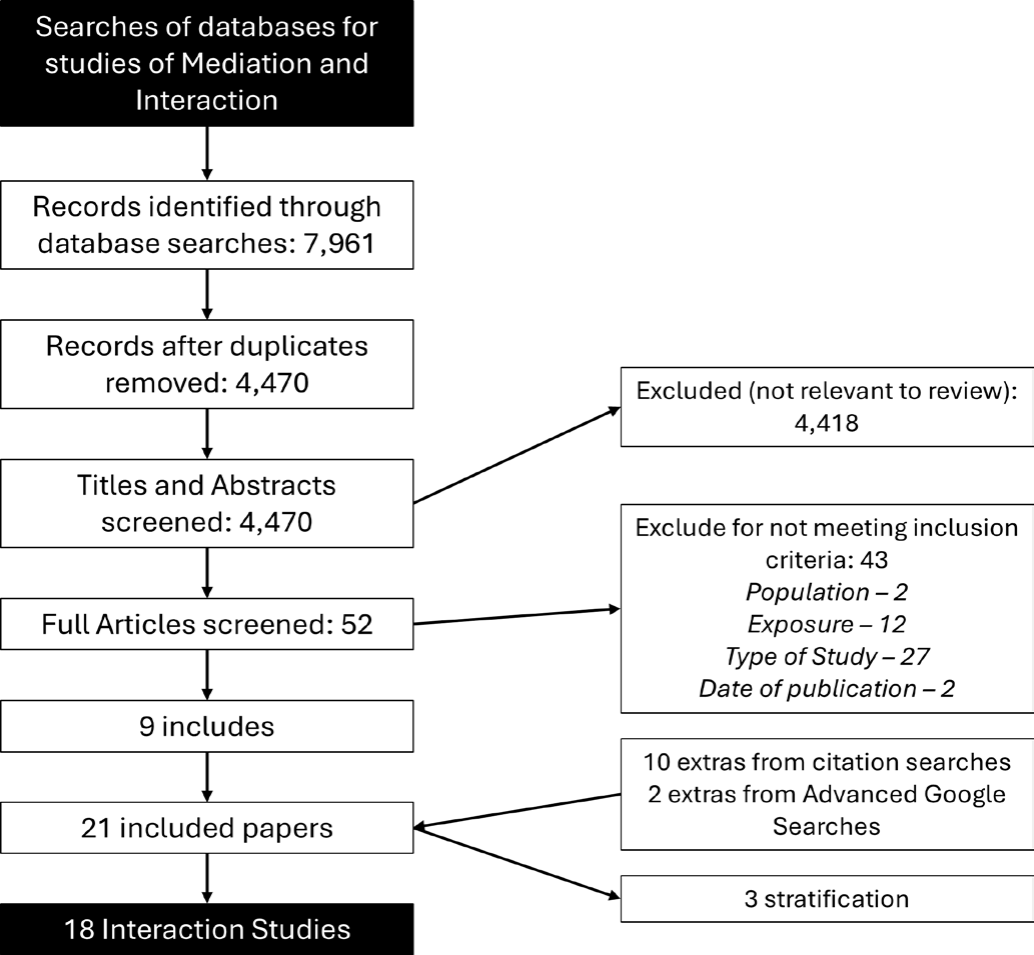
Preferred Reporting Items for Systematic Reviews and Meta-Analyses flow diagram for: ‘How does preterm birth influence the relationship between maternal socioeconomic status and adolescent outcomes for health and education?’

After removing duplicates, the initial searches identified 4470 papers to review, of which 52 were full-text screened (figure 1). Nine were included, and after screening and citation searches, a final total of 21 papers were included.^30^,^50^ Online supplemental appendix E shows the list of studies excluded at full text. Three of the studies did not calculate interaction and will be discussed separately.^30^,^32^ Of the 18 interaction studies, five were from Sweden,^37^,^4045^ four were from the USA,^34^,^3644^ three the UK^33 46 49^ and Netherlands each,^42 43 47^ and one each from Australia,^48^ Denmark^41^ and Italy (table 2).^50^ The earliest birth periods started in 1973 (four Swedish studies) and the latest birth period finished in 2016. All interaction studies were cohort design.

**Table 2:** Characteristics of included studies including country, birth period for the cohort, socioeconomic status (SES) indicator used, analysis approach and scale interaction are measured on

Paper	Country	Data source	Birth period	SES indicator	Analysis approach	Interaction
Beauregard *et al*^33^	UK	Birth Cohort Study	2000–2002	Household poverty measured at 9 months	GEE	Additive
Beauregard *et al*^34^	USA	Birth Cohort Study	2001	Household SES composite measured at 9 months	GEE	Additive
Mallinson *et al*^35^	USA	Linked Birth Cohort	2007–2010	Maternal education or maternal medicaid coverage measured at birth	Linear regression	NS
Richards *et al*^36^	USA	Linked Birth Cohort	1998–2002	Neighbourhood Deprivation Index measured at birth	GEE	Additive/multiplicative
Ekeus *et al*^37^	Sweden	National Registry	1973–1976	Dichotomised composite of parental education and occupation measured at 1990	Linear regression	Multiplicative
Lindström *et al*^38^	Sweden	National Registry	1973–1979	Household occupation measured at 1985	Logistic regression	NS
Lindström *et al*^39^	Sweden	National Registry	1973–1979	Composite household measure measured at 1985	Cox regression	NS
Bilsteen *et al*^41^	Denmark	National Registry	1982–1986	Maternal education measured prebirth	Multinomial logistic regression	NS
Gisselmann *et al*^40^	Sweden	Birth Cohort Study	1973–1981	Parents education measured at 1990	Logistic regression	NS
Potijk *et al*^42^	Netherlands	Birth Cohort Study	2002–2003	Composite measured at birth and at 4	Logistic regression	Multiplicative
Potijk *et al*^43^	Netherlands	Birth Cohort Study	2002–2003	Composite measured at birth and at 4	Logistic regression	Multiplicative
ElHassan *et al*^44^	USA	Birth Cohort Study	1998	Maternal education and maternal insurance coverage measured at birth	Linear mixed-effect models	Multiplicative
Lindström *et al*^45^	Sweden	National Registry	1987–2000	Education measured at 2005	Logistic regression	NS
Ene *et al*^46^	UK	Linked Birth Cohort	2011–2014	Quintile of Scottish Index of Multiple Deprivation	Logistic regression	Additive
de Laat *et al*^47^	Netherlands	Birth Cohort Study	2003–2004	Education and perceived income inadequacy measured at outcome	Linear regression	Multiplicative
Doyle *et al*^48^	Australia	Hospital Birth Cohort	1991–1992	Education and social class measured at 8	GEE	NS
Peacock *et al*^49^	UK	Birth Cohort Study	1991–1992	Social class	Logistic regression	NS
Dall'oglio *et al*^50^	Italy	Hospital Birth Cohort	1998–1999	Parental education measured At 4	Linear regression	NS

GEEgeneral estimating equationNSnot statedSESsocioeconomic status

### Methodological quality

Online supplemental appendix F shows the quality scoring for each study. Of the interaction studies, three potentially had selection bias,^35 47 50^ one potentially had response bias^47^ and eight potentially had follow-up bias.^3337 42 43 46^,^49^ Two studies used area-based measures of SES,^36 46^ which scored lower on quality than the rest, which used individual-based measures of SES. Four studies had potential measurement bias in PTB^4448^,^50^ while one had measurement bias for outcomes.^50^ Four studies made no adjustment for confounding or this was unclear,^42 43 46 49^ and six had extensive adjustment.^33^,^3644 47^ Seven studies reported both coefficient and significance estimates for the interaction term.^3335^,^37 42 43 46^

### Outcomes

**Figure 2:**
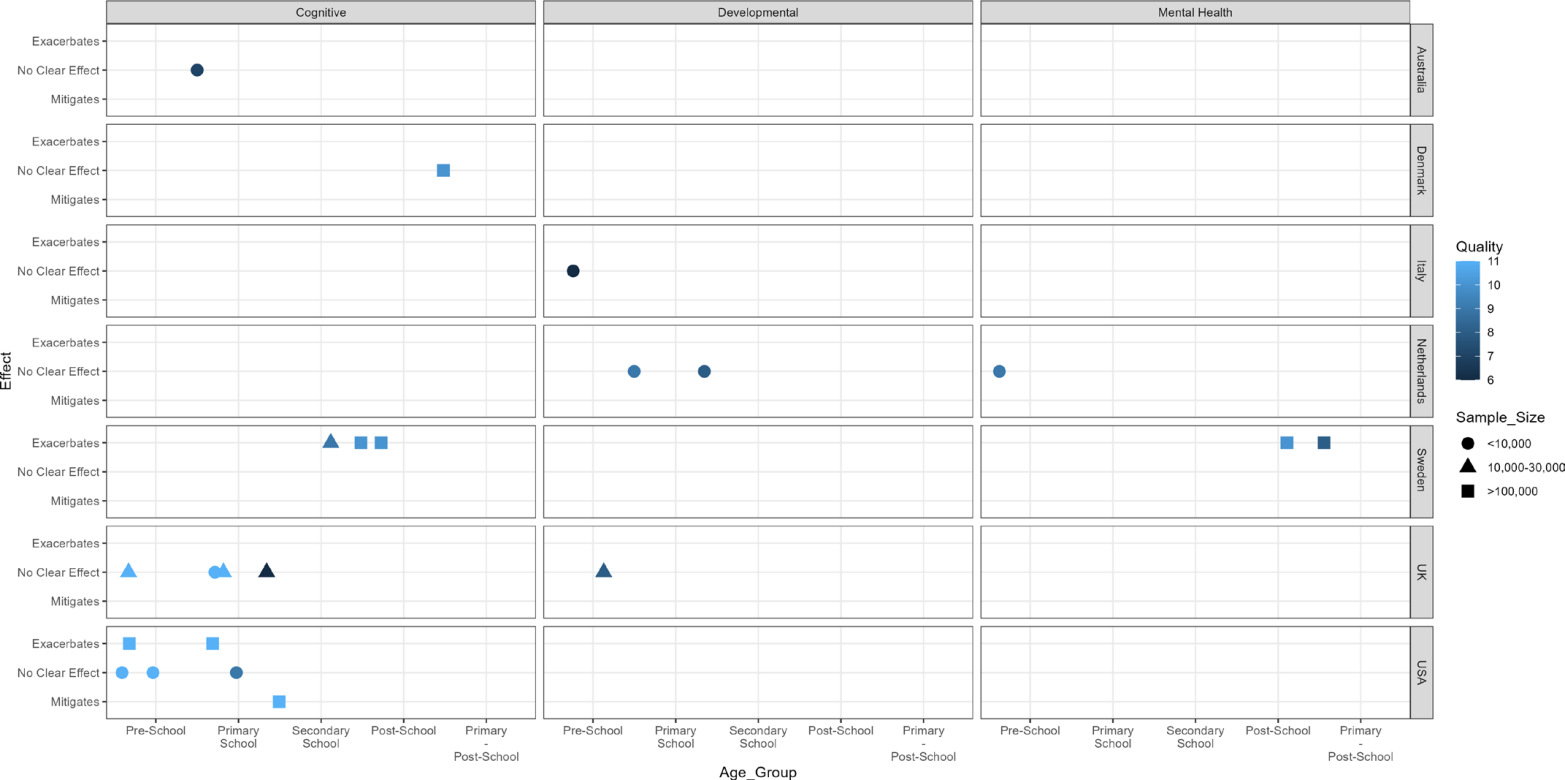
Harvest plot demonstrates the presence of a super- (exacerbates), sub- (mitigates), or no significant interaction (no clear effect) for three outcomes. Results split by country (right y-axis), age group (x-axis), sample size (shape) and quality (colour). Higher quality score means that study is of higher quality.

Figure 2 shows a harvest plot demonstrating significance and direction of interaction by outcomes, country, age group, sample size and study quality. Overall, 22 estimates were included from the 18 studies, as some studies included multiple measures and multiple age groups. Cognitive outcomes were the most examined outcome, and the youngest age groups were the most frequently studied (eight preschool and seven primary school). One study was in the harvest plot as it covered multiple age groups.^45^ Outcomes that do not fit into the categories have not been included in this plot. Table 3 shows the outcome, sample size and effects for each study.

**Table 3:** Interaction for each outcome by age group, sample size, quality score, effect of socioeconomic status (SES) and effect of preterm birth or gestational age (GA) for included studies

Paper	Age group	Sample size	Outcome	Effect of SES	Effect of preterm birth/GA	Interaction effect	QA
Beauregard *et al*^33^	Preschool	10 649	School readiness and vocabulary	Lower SES has significant association with lower scores	Lower GA has significant association with lower scores	No consistent pattern in either	11
	Primary school	10 494	Vocabulary and visuospatial ability	Lower SES has significant association with lower scores	Lower GA has significant association with lower scores for pattern construction	No consistent pattern in either	
	Primary school	9521	Reading, visuospatial ability and number skills	Lower SES has significant association with lower scores	Lower GA has significant association with lower scores in pattern construction and numeracy	No consistent pattern in either	
Beauregard *et al*^34^	Preschool	5250	Cognitive ability	Lower SES has significant association with lower scores	Lower GA has significant association with lower scores	No significant interaction	11
	Primary school	3800	Reading and maths	Lower SES has significant association with lower scores	Lower GA has significant association with lower scores	No significant interaction	
Mallinson *et al*^35^	Primary school	153 145	PALS-K and literacy	Lower SES has significant association with lower scores	Lower GA has significant association with lower scores	Significant interaction	11
Richards *et al*^36^	Primary school	327 698	Mathematics failure	Lower SES has significant association with increased risk of failure	Lower GA has significant association with increased risk of failure	Significant positive additive interaction/ significant negative multiplicative interaction/ RERI not significant	11
Ekeus *et al*^37^	Post school	119 664	Intelligence performance	Lower SES has significant association with lower scores	Lower GA has significant association with lower scores	Significant interaction	10
Lindström *et al*^38^	Post school	513 957	Cognitive	Not stated	Lower GA has significant association with lower rates of university	Significant positive interaction	10
	Post school	522 310	Disability	Not stated	Lower GA has significant association with increased disability	No significant interaction	
	Post school	380 168	Employment	Not stated	No significant differences	No significant interaction	
Lindström *et al*^39^	Post school	545 628	Psychiatric hospital admission	Not stated	Lower GA has significant association with increased odds of admission	Significant interaction	10
Bilsteen *et al*^41^	Post school	228 030	Educational attainment	Not stated	Lower GA has significant association with lower attainment	No significant interaction	10
			Income	Not stated	Lower GA has significant association with lower income	No significant interaction	
			Source of income	Not stated	Lower GA has significant association with higher rates of disability pension and welfare	No significant interaction	
Gisselmann *et al*^40^	Secondary school	10 242	School performance	Lower SES has significant association with lower acheivement	Lower GA has significant association with lower acheivement	Significant interaction	9
Potijk *et al*^42^	Preschool	1470	Total score	Lower SES has significant association with delay	No significant association	No significant interaction	9
			Fine motor	No significant association	Lower GA has significant association with delay	No significant interaction	
			Gross motor	No significant association	No significant association	No significant interaction	
			Communication	Lower SES has significant association with delay	Lower GA has significant association with delay	Significant interaction	
			Problem-solving	Lower SES has significant association with delay	No significant association	No significant interaction	
			Personal–social	Lower SES has significant association with delay	No significant association	No significant interaction	
Potijk *et al*^43^	Preschool	1458	Child Behaviour Checklist total problems	Lower SES has significant association with increased problems	Lower GA has significant association with increased problems	No significant interaction	9
			Externalising	No significant association	Lower GA has significant association with increased problems	No significant interaction	
			Internalising	Lower SES has significant association with increased problems	Lower GA has significant association with increased problems	No significant interaction	
ElHassan *et al*^44^	Primary–secondary school	1424	Literacy	Lower SES has significant association with lower scores	Lowest GA has significant association with lower scores	No significant interaction	9
			Mathematics	Lower SES has significant association with lower scores	Lowest GA has significant association with lower scores	No significant interaction	
Lindström *et al*^45^	Primary–post school	1 180 616	ADHD medication prescription	Not stated	Lower GA has significant association with increased odds of prescription	Significant interaction	8
Ene *et al*^46^	Preschool	28 634	ASQ-3—speech and language concerns	No significant association	Lower GA has significant association with increased odds of concerns	No significant interaction	8
de Laat *et al*^47^	Primary school	4451	SDQ total difficulties score—mother	Not stated	Lower GA has significant association with higher problems	No significant interaction	8
		3580	SDQ total difficulties score—teacher	Not stated	No significant association	No significant interaction	
Doyle *et al*^48^	Primary school and secondary school	560	Reading, spelling and maths	Lower SES has significant association with lower scores	Lower GA has significant association with lower scores at age eight only	No significant interaction	7
Peacock *et al*^49^	Primary school	10 260	KS1 score	Not stated	Lower GA has significant association with lower success	No significant interaction	6
Dall'oglio *et al*^50^	Preschool	85	Griffiths Mental Developmental Scales	Lower SES has significant association with lower scores	Lower GA has significant association with lower scores	No significant interaction	6

ADHDAttention Deficit Hyperactivity DisorderASQAges and Stages QuestionnaireKS1Key Stage 1PALS-KPhonological Awareness Literacy Screening for KindergartenQAquality appraisal scoreRERIrelative excess risk due to interactionSDQStrengths and Difficulties Questionnaire

#### Cognitive

Eleven studies examined cognitive outcomes, with 15 interaction estimates (one study used three age groups). The first study by Beauregard *et al*^33^ used evidence from a UK birth cohort study to estimate the effect of household poverty (income less than 60% of the median) and GA at birth categories (early/moderate PTB (24–33 weeks), late preterm (34–36), early term (37–38) and term (39–40)) on various educational outcomes at ages 3 (school readiness and naming vocabulary), 5 (naming vocabulary, picture similarity and pattern construction), and 7 years (word reading, pattern construction and number skills). Household poverty was significantly associated with lower scores for all outcomes in all age groups in interaction models. PTB categories were significantly associated with lower scores for school readiness (not early term) and naming vocabulary (only early/moderate PTB) at age 3, pattern construction at ages 5 and 7, and number skills at age 7 (for early/moderate PTB only). The interaction between early/moderate PTB and poverty for word reading at age 7 was superadditive. The interaction between early term and poverty for pattern construction at age 7 had a subadditive effect. No other significant interactions were found, suggesting no overall clear pattern was noted; the effect of the two exposures was not modified by the other.

The second study by Beauregard *et al*^34^ used evidence from a USA population-based birth cohort study to estimate the effect of a composite measure of SES (parental education, occupation and income) and lower GA on cognitive ability at age 2, and reading and mathematics at age 6 (Kindergarten). Both decreasing SES and earlier GA groups had significant proportional negative effects on cognitive ability (at age 2) and both reading and mathematics assessment. No significant interactions between GA and SES were found, and direction of estimated interaction effect was not noted.

Mallinson *et al*^35^ used data on all births in Wisconsin to estimate the effects of maternal Medicaid coverage (government-provided health insurance), maternal education and GA (as a continuous variable) on standardised testing and passing literacy benchmark at kindergarten age. Lower maternal education and Medicaid coverage were both significantly associated with lower scores and not passing benchmark as was decreasing GA. Interaction between Medicaid or education and GA was significant (interaction scale not stated) and suggested lower SES exacerbated effects of lower GA on outcomes when harmonised.

Richards *et al*^36^ used data from a retrospective birth cohort in Georgia, USA to estimate the effects of neighbourhood deprivation and PTB on mathematics failure during first grade. PTB and increasing deprivation were both associated with significantly increased risk of failure. Significant superadditive and submultiplicative interactions were seen, while the relative excess risk due to interaction (RERI) was not significant. This suggests two potential scenarios, exposure to both PTB and low SES exacerbate the negative effects of each and the RERI calculation was underpowered, or the superadditive interaction is a spurious finding.

Ekeus *et al*^37^ used Swedish register data to estimate the effects of a composite measure of SES and PTB on intelligence performance in 18-year-old to 19-year-old young men at conscription. Lower SES and PTB were both associated with significantly lower performance, while a significant supermultiplicative interaction was found for moderate PTB (33–36 weeks) only.

Lindström *et al*^38^ used Swedish register data to estimate the effects of household occupation and PTB on postsecondary education in 23–29 years old. Lower GA groups had significantly lower rates of postsecondary education and a significant positive interaction (type not stated) with household occupation was found; low SES exacerbated the effects of PTB.

Bilsteen *et al*^41^ used Danish register data to estimate the effects of maternal education and GA on educational attainment at age 28. Lower GA was associated with significantly lower odds of tertiary or secondary education and no significant interaction was seen. The effect of maternal education was not stated.

Gisselmann *et al*^40^ used Swedish register data to estimate the effects of parental education and PTB on school performance at ages 15–16. Both lower education and PTB were associated with lower achievement, and significant interaction demonstrated that low SES exacerbated the effect of PTB (interaction scale not stated).

ElHassan *et al*^44^ used data on births in Arkansas, USA, to estimate the effect of low SES (using maternal education and maternal insurance coverage measured at birth) and GA groups on maths and literacy attainment between the third and eighth grades (8–14 years old). Both low SES and extremely low GA group were associated with significantly lower scores in both subjects, but no significant multiplicative interaction was seen (the effects of SES are the same in all GA groups, they are multiplicative).

Doyle *et al*^48^ conducted an Australian study using data on consecutive extremely preterm or extremely low birth weight births born during the study period who survived, that looked at the effects of education or social class and PTB on reading, spelling and maths at ages 8 and 18. Lower GA and low SES were associated with worse scores at age 8, and only low SES at age 18. No significant interaction was found.

Peacock *et al*^49^ used data from a UK-based cohort study to estimate the effect of social class and GA on Key Stage 1 (KS1) scores (ages 5–7). Children born preterm were less likely to attain KS1 and specific areas (reading, writing and mathematics). No significant interaction was found.

#### Developmental

Four studies examined the interaction between SES and GA on developmental outcomes. Potijk *et al*^42^ used a prospective cohort from the Netherlands to estimate the effects of a composite measure of SES and GA on developmental delay at age 4. Decreasing SES and GA were both significant for increasing/worsening communication delay, and submultiplicative interaction was seen. This means that the relative effect of having both low SES and PTB on communication delay is lower than if the risks of both were multiplied. For other developmental outcomes (total score, fine motor, gross motor, problem-solving, personal–social), interaction was multiplicative (the effect of both risks is the same as each single risk combined). Notably, none of these outcomes had both risks significant independently. Shown on figure 2 as no clear effect.

Ene *et al*^46^ used a record-based cohort to examine the effects of area-based deprivation and PTB on speech and language concerns at age 2.5. Lower GA was associated with higher levels of concern, while SES was not associated. No significant additive interaction was found.

de Laat *et al*^47^ used data from a Dutch population-based cohort to estimate effects of PTB and two measures of SES (maternal education and perceived income inadequacy) on total difficulties at ages 5–6 assessed by mothers and teachers. Difficulties were more common in preterm children on maternal assessment. No significant multiplicative interaction was found.

Dall’oglio *et al*^50^ used data from a very small Italian cohort study to examine the effects PTB categories and parental education on Griffiths Mental Developmental Scales at age 4. Both PTB and low SES were significantly associated with worse scores. No significant interaction was found.

#### Mental health

Three studies examined interaction between GA and SES on mental health outcomes. Lindström *et al*^39^ used Swedish register data to estimate the effects on hospital admissions for a psychiatric diagnosis by age 23–29. The effect of SES was not stated; however, lower GA was significantly associated with higher odds of admission. A significant interaction was found; lower SES exacerbated the GA impacts (scale for interaction not stated).

Potijk *et al*^43^ use a Dutch population-based cohort to estimate the effects on total behavioural problems, internalising problems and externalising problems. Low SES and PTB were both associated with more total and internalising problems, while externalising problems were only associated with PTB. No significant interaction was found; all combinations were multiplicative.

Lindström *et al*^45^ used Swedish register data to estimate the effects of SES and PTB category on ADHD medication prescription from age 6 through to 19. A significant interaction was found; the effect of moderate PTB was exacerbated by low SES.

#### Other

Lindström *et al*^38^ estimated the effects of household occupation and PTB on disability and unemployment in 23–29 years old. No significant interaction was found. Bilsteen *et al*^41^ estimated the effects of maternal education and GA on income tertile and source of income. Lower GA was significantly associated with lower odds of being in the highest income tertile, and higher odds of getting income from cash benefits and disability pension. There was no significant interaction found.

##### Sample size

We split the sample sizes into three broad groups; less than 10 000 (eight studies), between 10 000 and 30 000 (four studies), and over 100 000 (seven studies). One study provided estimates for two sample size categories.^33^ Of the studies that found a significant interaction between SES and GA, one had a sample size in the less than 10 000 range (for developmental outcomes),^42^ one was between 10 000 and 30 000 (for cognitive outcomes)^40^ and the other six studies were all over 100 000.^35^,^3945^ Two studies with a sample over 100 000 found no significant interaction for assorted outcomes post school.^38 41^ The study with less than 10 000 participants only found a significant interaction for one out of five outcomes.^42^

##### Confounders

The most frequently adjusted confounder was sex of the child (15/18). Other variables included child age at outcome or year of birth (9), number of siblings or maternal parity (8), maternal age at birth (7), ethnicity or race (6), and number of parents or parental/maternal marital status (6). Birth weight, multiple gestation, Apagar score (measured at birth), smoking during pregnancy and gestational diabetes were sporadically included.

##### Stratification papers and other methods

Baranowska-Rataj *et al*^32^ used Swedish register data (1 087 750) to determine the effect of PTB and parental education/occupation/income quintile separately on grades in the final year of compulsory schooling, compared with non-preterm siblings. Worse grade outcomes were observed for very and extremely preterm groups. When combining the two exposures (dummy variables which combine PTB category and SES), there were no clear differences in the effects.

Kroll *et al*^30^ used routine UK data to estimate the effects of area-based deprivation and GA groups on infant mortality. For children born at less than 32 weeks gestation, there were no socioeconomic inequalities in all-cause mortality. For those born between 32 and 37 weeks, low SES was associated with higher all-cause mortality.

Ray *et al*^31^ used a Canadian population-based cohort study to estimate the effects on neonatal mortality. PTB significantly increased risk and stratification by income quintile and did not change the pattern.

##### Updated searches

Searches were rapidly updated, and single screened, in Scopus and advanced Google up until July 2023. A small UK study found that GA and area-based SES had no interaction but GA and family-based measures of SES had some significant interactive effects on brain structure measures.^51^ A large Danish register-based study found a superadditive interaction between GA and SES on the risk of not completing schooling at 15–16 years old.^52^ A small study from USA found no significant interaction between PTB and social adversity on executive function.^53^ These studies are in keeping with our current findings.

## Discussion

### Principal findings

We conducted a systematic review of interaction and effect modification of SES and low GA or PTB on child and adolescent outcomes. Most of the studies focused on interaction (18/21). We found that low SES and low GA are consistently associated with worse cognitive, developmental and mental health outcomes. For both cognitive outcomes and mental health outcomes, we found evidence from the largest studies of a superadditive or supermultiplicative interaction, and low GA exacerbated the negative effects of low SES (or vice versa). Only one study found a significant interaction of SES and GA on developmental outcomes, with a submultiplicative effect for communication delay—the relative effect of having both exposures together is lower than having both risks separately.

Notably, the significance of interaction terms seemed to be linked with study sample size; only two (of seven) studies with a sample over 100 000 found no significant interaction and only two smaller studies found a significant interaction. Given the large sample sizes required for interaction analyses to be adequately powered, it is possible that some of the studies with null effects were underpowered.^23^

The two large studies that did not detect a significant interaction were focused on outcomes measured post school, for disability, income, employment and educational attainment. Conversely, three estimates found a significant superinteraction effect in this age group for cognitive measures and psychiatric morbidity. This suggests that the interaction effects are outcome dependent—mental health and cognitive effects are exacerbated even through to post school period. Country may also impact these findings (eg, through social support programmes); however, no clear pattern was seen for these estimates. Our ability to make further comment on the effects of other contrasts (including country) is limited by the effects of sample size. The effects of a potential interaction by age at which the outcome occurs, and the measure of SES would merit investigation.

### Relevance to other studies

The findings of this review suggest that children born preterm are more susceptible to the negative consequences of low SES for cognitive and mental health outcomes (differential susceptibility), or the consequences of being born preterm are exacerbated by low SES (differential disease consequences). This is an important finding when considered alongside the increased risk of PTB in low SES groups,^4^ meaning the cohort of children exposed to both these risks will be larger, and thus the potential to exacerbate inequalities is increased.

Interaction between SES and health has been shown to influence outcomes. For example, SES has been shown to have a superadditive interaction with maternal mental health on PTB,^54^ while low birth weight has been shown to have a superadditive interaction with unhealthy lifestyle on type 2 diabetes.^55^ This has implications for understanding the mechanisms that generate health inequalities, specifically differential susceptibility and differential disease consequences as described in the Diderichsen model of health inequalities.^17^

### Strengths

Our study used extensive searches of multiple databases and supplementary searches, so we are confident we have identified most relevant studies. Additionally, we specifically considered interaction in our methodological quality appraisal, increasing the relevance of the quality scores to our study.

### Limitations of the review

Some studies in our review included interaction as an additional element to the analysis. It is therefore possible that we may have missed some papers because titles and abstracts were screened for those mentioning interaction or stratification. If not mentioned, studies did not make it through to full-text screening, and therefore some valid studies may potentially have been missed. Given this, our focus on supplementary search strategies is valuable to comprehensively identifying relevant evidence. Such an approach has been applied previously to identify hard-to-find evidence on health inequalities.^27 56 57^ Additionally, interaction and effect modification are similar but distinct concepts, thus comparison of the two may not be appropriate; however, this is unlikely to have influence our study as most studies focused on interaction.^16^

### Limitations of the data

There are some limitations that should be considered when interpreting this evidence. First, it should be noted that most of the studies that found no significant interaction were smaller sample size. Studies that aim to find interaction need a very large sample size to be adequately powered to identify an effect. Therefore, the non-significant findings are potentially due to underpowered studies and cannot be interpreted as evidence of no interaction between SES and low GA.

A second limitation was the reporting of interaction effects. Half the interaction studies did not state the scale interaction was measured on, and 11 did not report both significance level and the coefficient. Understanding whether interaction is measured on the multiplicative scale or the additive scale is important for interpretation, with results measured on the additive scale being more relevant to public health.^15^ Richards *et al* demonstrate this, as the direction of effect can reverse based on scale.^36^

A final limitation is around the lack of studies examining health outcomes. Both exposures are associated with worse health outcomes, and there is evidence that SES impacts healthcare usage in preterm children.^58^ The lack of studies that have examined the interplay between SES and PTB on health outcomes is a notable gap.

### Conclusions

Our findings suggest that the harmful impacts of PTB and low SES on childhood cognitive and mental health outcomes are exacerbated by the other risk, generating health inequalities. Children who are born into low SES are more susceptible to the negative cognitive and mental health consequences of PTB and vice versa. They would benefit from additional support to fully achieve their educational potential and reduce adolescent mental health problems.^59 60^ Early interventions for children born preterm should include parents and psychosocial support to maximise development. Action to alleviate the negative effects of low SES, reduce PTB prevalence and target children with both exposures is indicated to reduce socioeconomic inequalities.^61^

## supplementary material

10.1136/bmjopen-2024-084147 online supplemental file 1

10.1136/bmjopen-2024-084147 online supplemental file 2

10.1136/bmjopen-2024-084147 online supplemental file 3

10.1136/bmjopen-2024-084147 online supplemental file 4

10.1136/bmjopen-2024-084147 online supplemental file 5

10.1136/bmjopen-2024-084147 online supplemental file 6

## Data Availability

All data relevant to the study are included in the article or uploaded as supplementary information.
