# Supplementary material for: How do socioeconomic inequalities and preterm birth interact to modify health and education outcomes? A narrative systematic review
Source: BMJ Open. 2025 Jan 25;15(1):e084147. doi: 10.1136/bmjopen-2024-084147 (PMC11784320; doi:10.1136/bmjopen-2024-084147)
Supplement: online supplemental file 2 [file bmjopen-15-1-s002.docx]

# Appendix B – Changes to the PROSPERO protocol

There were three minor changes from our protocol:

- We double-screened 15% of titles and abstracts rather than 20%. When we calibrated at 15%, agreement was high (over 99%) between the two reviewers, and further double-screening was not required.
- The search strategy was expanded and further detailed in the manuscript. A detailed search strategy was not included in the protocol.
- The Liverpool Quality Assessment tool was modified to appraise quality in the context of interaction. We used this rather than the Newcastle-Ottawa tool as it covered the areas we identified in the protocol plus interaction quality in a straightforward way.

These minor changes have not impacted on our findings or introduced a new risk of bias.
