# Supplementary material for: How do socioeconomic inequalities and preterm birth interact to modify health and education outcomes? A narrative systematic review
Source: BMJ Open. 2025 Jan 25;15(1):e084147. doi: 10.1136/bmjopen-2024-084147 (PMC11784320; doi:10.1136/bmjopen-2024-084147)
Supplement: online supplemental file 3 [file bmjopen-15-1-s003.docx]

# Appendix C – Search Strategy

The included search terms were:

1. mediat* OR (structural equation modelling) OR (structural equation modelling) OR path OR (Baron and Kenny) OR MacKinnon OR (product of coefficient) OR (difference in coefficient) OR (process of change) OR sobel* OR (causal pathway) OR intermediate OR (process variable) OR (treatment ADJ2 effect) OR (process ADJ2 evaluation) OR mechanism OR SEM OR modifi* or interact* or (differential exposure) or (differential susceptibility) or attenuat* or indirect* or decomp*
2. disparit* OR socio-economic OR socioeconomic OR depriv* OR inequal* OR poverty OR inequit* OR disadvantag*
3. preterm OR prematur* OR gestation
4. 1 AND 2 AND 3

Search terms were consistent across all databases. Only the operators varied, as required for each database. The same search terms were used for Advanced Google search, however limitations in number of terms required the search to split into five sub-searches, as follows:

- (“mediat*” | “structural equation modelling” | “path” | “attenuat*” | “indirect*” | “decomp*”) (“disparit*” | “socioeconomic” | “depriv*” | “inequal*” | “poverty” | “inequit*” | “disadvantag*”) (“preterm” | “prematur*” | “gestation*”)
- (“Baron and Kenny” | “MacKinnon” | “product of coefficient” | “difference in coefficient” ) (“disparit*” | “socioeconomic” | “depriv*” | “inequal*” | “poverty” | “inequit*” | “disadvantag*”) (“preterm” | “prematur*” | “gestation*”)
- (“process of change” | “sobel*” | “causal pathway” | “intermediate” | “process variable”) (“disparit*” | “socioeconomic” | “depriv*” | “inequal*” | “poverty” | “inequit*” | “disadvantag*”) (“preterm” | “prematur*” | “gestation*”)
- (“treatment ADJ2” | “process ADJ2 evaluation” | “mechanism” | “SEM” | “modifi*” | “interact*”) (“disparit*” | “socioeconomic” | “depriv*” | “inequal*” | “poverty” | “inequit*” | “disadvantag*”) (“preterm” | “prematur*” | “gestation*”)
- (“differential exposure” | “differential susceptibility”) (“disparit*” | “socioeconomic” | “depriv*” | “inequal*” | “poverty” | “inequit*” | “disadvantag*”) (“preterm” | “prematur*” | “gestation*”)

The first ten pages for each search were screened.

**Updated searches** - Scopus

1. Modif* or interact* or (differential exposure) or (differential susceptibility) or attenuat* or indirect* or decomp*
2. disparit* OR socio-economic OR socioeconomic OR depriv* OR inequal* OR poverty OR inequit* OR disadvantag*
3. preterm OR prematur* OR gestation
4. 1 AND 2 AND 3
