# Supplementary material for: How do socioeconomic inequalities and preterm birth interact to modify health and education outcomes? A narrative systematic review
Source: BMJ Open. 2025 Jan 25;15(1):e084147. doi: 10.1136/bmjopen-2024-084147 (PMC11784320; doi:10.1136/bmjopen-2024-084147)
Supplement: online supplemental file 4 [file bmjopen-15-1-s004.docx]

# Appendix D – Quality Appraisal

Selection

This was measured by three domains, each scoring 1 point

- Selection bias: was there evidence in selection of the sample that may introduce bias. If no – 1, if yes/possible - 0
- Response bias: was there evidence of systematic difference between responders and non-responders. If no – 1, if yes/possible - 0
- Follow-up bias: was there evidence of loss to follow-up and did those lost differ. If no – 1, if yes/possible - 0

Measurement

This was measured in four domains:

- SES measure: if individual based score - 2, if area-based score - 1
- Measurement bias for preterm birth: if estimated clearly (e.g. from last menstrual period) - 1
- Ascertainment/measurement bias of outcome: if no evidence, score of 1 for each to a maximum of 2

Confounding

Assessed to a max of 2. No or limited adjustment - 0, adequate adjustment - 1, comprehensive adjustment – 2. Adequate or comprehensive adjustment was agreed by the two reviewers and considered the inclusion of demographic, health and social covariates. Failure to adjust for all demographic variables excluded a study from a potential maximum score.

Interaction

Interaction result was assessed to a max of 2. If mentioned in text as simply significant/non-significant – 0, if either coefficient or measure of statistical significance was included – 1, if both included – 2
