# Supplementary material for: How do socioeconomic inequalities and preterm birth interact to modify health and education outcomes? A narrative systematic review
Source: BMJ Open. 2025 Jan 25;15(1):e084147. doi: 10.1136/bmjopen-2024-084147 (PMC11784320; doi:10.1136/bmjopen-2024-084147)
Supplement: online supplemental file 5 [file bmjopen-15-1-s005.docx]

**Appendix E – Table showing studies excluded at full-text screening with reason for exclusion**

| **Exclude due to Population** |
| --- |
| Dilworth-Bart JE, Poehlmann JA, Miller KE, Hilgendorf AE. Do mothers’ play behaviors moderate the associations between socioeconomic status and 24-month neurocognitive outcomes of toddlers born preterm or with low birth weight?. Journal of Pediatric Psychology. 2011 Apr 1;36(3):289-300. |
| Sentenac M, Johnson S, Charkaluk ML, Sëppanen AV, Aden U, Cuttini M, Maier R, Mannamaa M, Zeitlin J. Maternal education and language development at 2 years corrected age in children born very preterm: results from a European population-based cohort study. J Epidemiol Community Health. 2020 Apr 1;74(4):346-53. |
| **Exclude due to Exposure** |
| Schneider W, Wolke D, Schlagmüller M, Meyer R. Pathsways to school achievement in very preterm and full term children. European Journal of Psychology of Education. 2004 Dec;19(4):385-406. |
| Amutah NN. Neighborhood Level Disadvantage, Race/Ethnicity and Infant Mortality in Washington DC. |
| Eriksson JG, Salonen MK, Kajantie E, Osmond C. Prenatal growth and CKD in older adults: longitudinal findings from the Helsinki birth cohort study, 1924-1944. American Journal of Kidney Diseases. 2018 Jan 1;71(1):20-6. |
| Ford RM, Neulinger K, O'Callaghan M, Mohay H, Gray P, Shum D. Executive function in 7–9-year-old children born extremely preterm or with extremely low birth weight: effects of biomedical history, age at assessment, and socioeconomic status. Archives of Clinical Neuropsychology. 2011 Nov 1;26(7):632-44. |
| Harbor RL. Predicting school behavior problems and delinquency from birth information: The role of intermediate risk and protective factors. University of South Florida; 2000. |
| Hitzert MM, Van Braeckel KN, Bos AF, Hunnius S, Geuze RH. Early visual attention in preterm and fullterm infants in relation to cognitive and motor outcomes at school age: an exploratory study. Frontiers in pediatrics. 2014 Oct 6;2:106. |
| Kelly MM, Li K. Poverty, toxic stress, and education in children born preterm. Nursing research. 2019 Jul 1;68(4):275-84. |
| Kull M. *Early physical health problems as developmental liabilities for school readiness: Associations with early learning contexts and family socioeconomic status* (Doctoral dissertation, Doctoral Dissertation. Boston College. 2015. Available from: http://hdl. handle. net/2345/bc-ir: 104143). |
| Kull MA, Coley RL. Early physical health conditions and school readiness skills in a prospective birth cohort of US children. Social Science & Medicine. 2015 Oct 1;142:145-53. |
| Leung JY, Lam HS, Leung GM, Schooling CM. Gestational age, birthweight for gestational age, and childhood hospitalisations for asthma and other wheezing disorders. Paediatric and perinatal epidemiology. 2016 Mar;30(2):149-59. |
| Vederhus BJ, Markestad T, Eide GE, Graue M, Halvorsen T. Health related quality of life after extremely preterm birth: a matched controlled cohort study. Health and quality of life outcomes. 2010 Dec;8:1-8. |
| Wang WL, Sung YT, Sung FC, Lu TH, Kuo SC, Li CY. Low birth weight, prematurity, and paternal social status: impact on the basic competence test in Taiwanese adolescents. The Journal of pediatrics. 2008 Sep 1;153(3):333-8. |
| **Exclude due to Type of Study** |
| Alnæs D, Kaufmann T, Marquand AF, Smith SM, Westlye LT. Patterns of sociocognitive stratification and perinatal risk in the child brain. Proceedings of the National Academy of Sciences. 2020 Jun 2;117(22):12419-27. |
| Andersson AK, Martin L, Brodd KS, Almqvist L. Predictors for everyday functioning in preschool children born preterm and at term. Early Human Development. 2016 Dec 1;103:147-53. |
| Andersson AK, Martin L, Brodd KS, Almqvist L. Patterns of everyday functioning in preschool children born preterm and at term. Research in Developmental Disabilities. 2017 Aug 1;67:82-93. |
| Androutsos O, Moschonis G, Ierodiakonou D, Karatzi K, De Bourdeaudhuij I, Iotova V, Zych K, Moreno LA, Koletzko B, Manios Y, ToyBox study Group. Perinatal and lifestyle factors mediate the association between maternal education and preschool children's weight status: the ToyBox study. Nutrition. 2018 Apr 1;48:6-12. |
| Anthopolos R, Edwards SE, Miranda ML. Effects of Maternal Prenatal Smoking and Birth Outcomes Extending into the Normal Range on Academic Performance in Fourth Grade in North C arolina, USA. Paediatric and perinatal epidemiology. 2013 Nov;27(6):564-74. |
| Benzies KM, Harrison MJ, Magill‐Evans J. Parenting stress, marital quality, and child behavior problems at age 7 years. Public Health Nursing. 2004 Mar;21(2):111-21. |
| Bouthoorn SH, Wijtzes AI, Jaddoe VW, Hofman A, Raat H, van Lenthe FJ. Development of socioeconomic inequalities in obesity among Dutch pre‐school and school‐aged children. Obesity. 2014 Oct;22(10):2230-7. |
| Butcher PR, Bouma A, Stremmelaar EF, Bos AF, Smithson M, Van Braeckel KN. Visuospatial perception in children born preterm with no major neurological disorders. Neuropsychology. 2012 Nov;26(6):723. |
| Caixeta HC, Amato AA. Factors associated with overweight and abdominal obesity in Brazilian school-aged children: a comprehensive approach. Archives of Endocrinology and Metabolism. 2020 Apr 6;64(4):445-53. |
| Calhoun SL, Vgontzas AN, Mayes SD, Tsaoussoglou M, Sauder K, Mahr F, Karippot A, Wisner K, Bixler EO. Prenatal and perinatal complications: is it the link between race and SES and childhood sleep disordered breathing?. Journal of clinical sleep medicine. 2010 Jun 15;6(3):264-9. |
| Conrad AL, Richman L, Lindgren S, Nopoulos P. Biological and environmental predictors of behavioral sequelae in children born preterm. Pediatrics. 2010 Jan 1;125(1):e83-9. |
| Crockett LK, Brownell MD, Heaman MI, Ruth CA, Prior HJ. Examining early childhood health outcomes of children born late preterm in urban Manitoba. Maternal and Child Health Journal. 2017 Dec;21:2141-8. |
| Dalziel SR, Lim VK, Lambert A, McCarthy D, Parag V, Rodgers A, Harding JE. Psychological functioning and health‐related quality of life in adulthood after preterm birth. Developmental Medicine & Child Neurology. 2007 Aug;49(8):597-602. |
| Delobel-Ayoub M, Arnaud C, White-Koning M, Casper C, Pierrat V, Garel M, Burguet A, Roze JC, Matis J, Picaud JC, Kaminski M. Behavioral problems and cognitive performance at 5 years of age after very preterm birth: the EPIPAGE Study. Pediatrics. 2009 Jun 1;123(6):1485-92. |
| Delobel-Ayoub M, Kaminski M, Marret S, Burguet A, Marchand L, N′ Guyen S, Matis J, Thiriez G, Fresson J, Arnaud C, Poher M. Behavioral outcome at 3 years of age in very preterm infants: the EPIPAGE study. Pediatrics. 2006 Jun 1;117(6):1996-2005. |
| D’Onofrio BM, Class QA, Rickert ME, Larsson H, Långström N, Lichtenstein P. Preterm birth and mortality and morbidity: a population-based quasi-experimental study. JAMA psychiatry. 2013 Nov 1;70(11):1231-40. |
| Foster-Cohen SH, Friesen MD, Champion PR, Woodward LJ. High prevalence/low severity language delay in preschool children born very preterm. Journal of Developmental & Behavioral Pediatrics. 2010 Oct 1;31(8):658-67. |
| Gullo DF, Miller M. Building a structural model for understanding school readiness. European Early Childhood Education Research Journal. 2018 Sep 3;26(5):718-37. |
| Hillemeier MM, Farkas G, Morgan PL, Martin MA, Maczuga SA. Disparities in the prevalence of cognitive delay: how early do they appear?. Paediatric and perinatal epidemiology. 2009 May;23(3):186-98. |
| Hutchinson EA, De Luca CR, Doyle LW, Roberts G, Anderson PJ, Victorian Infant Collaborative Study Group. School-age outcomes of extremely preterm or extremely low birth weight children. Pediatrics. 2013 Apr 1;131(4):e1053-61. |
| Johnson S, Waheed G, Manktelow BN, Field DJ, Marlow N, Draper ES, Boyle EM. Differentiating the preterm phenotype: distinct profiles of cognitive and behavioral development following late and moderately preterm birth. The Journal of pediatrics. 2018 Feb 1;193:85-92. |
| Jones KM, Champion PR, Woodward LJ. Social competence of preschool children born very preterm. Early human development. 2013 Oct 1;89(10):795-802. |
| Larranaga I, Santa-Marina L, Molinuevo A, Alvarez-Pedrerol M, Fernandez-Somoano A, Jimenez-Zabala A, Rebagliato M, Rodríguez-Bernal CL, Tardon A, Vrijheid M, Ibarluzea J. Poor mothers, unhealthy children: the transmission of health inequalities in the INMA study, Spain. European Journal of Public Health. 2019 Jun 1;29(3):568-74. |
| Playford CJ, Dibben C, Williamson L. Socioeconomic disadvantage, fetal environment and child development: linked Scottish administrative records based study. International journal for equity in health. 2017 Dec;16:1-3. |
| Reilly S, Wake M, Ukoumunne OC, Bavin E, Prior M, Cini E, Conway L, Eadie P, Bretherton L. Predicting language outcomes at 4 years of age: findings from Early Language in Victoria Study. Pediatrics. 2010 Dec 1;126(6):e1530-7. |
| Taylor-Robinson DC, Pearce A, Whitehead M, Smyth R, Law C. Social inequalities in wheezing in children: findings from the UK Millennium Cohort Study. European Respiratory Journal. 2016 Mar 1;47(3):818-28. |
| Yu Y, Liew Z, Wang A, Arah OA, Li J, Olsen J, Cnattingius S, Qin G, Obel C, Fu B, Li J. Mediating roles of preterm birth and restricted fetal growth in the relationship between maternal education and infant mortality: A Danish population-based cohort study. PLoS medicine. 2019 Jun 14;16(6):e1002831. |
| **Exclude due to date** |
| Berg CR. Parent interactions with fullterm and preterm preschoolers. Washington University in St. Louis; 1999. |
| Fan AP. The influence of perinatal complications and early social environment on mental health and status attainment in adulthood: The Baltimore NCPP follow-up, 1960–1994. The Johns Hopkins University; 2000. |
