# Supplementary material for: How do socioeconomic inequalities and preterm birth interact to modify health and education outcomes? A narrative systematic review
Source: BMJ Open. 2025 Jan 25;15(1):e084147. doi: 10.1136/bmjopen-2024-084147 (PMC11784320; doi:10.1136/bmjopen-2024-084147)
Supplement: online supplemental file 6 [file bmjopen-15-1-s006.docx]

**Appendix F – Table showing quality appraisal score for each study; Conf.=Confounding, Int.=Interaction**

| **Author** | **Selection bias** | **Response bias** | **Bias follow-up** | **SES** | **Measurement bias PTB** | **Ascertainment bias** | **Measurement bias in outcome** | **Conf.** | **Int.** | **Total Score** |
| --- | --- | --- | --- | --- | --- | --- | --- | --- | --- | --- |
| Beauregard *et al*.^31^ | 1 | 1 | 0 | 2 | 1 | 1 | 1 | 2 | 2 | 11 |
| Beauregard *et al*.^32^ | 1 | 1 | 1 | 2 | 1 | 1 | 1 | 2 | 1 | 11 |
| Mallinson *et al*.^33^ | 0 | 1 | 1 | 2 | 1 | 1 | 1 | 2 | 2 | 11 |
| Richards *et al*.^34^ | 1 | 1 | 1 | 1 | 1 | 1 | 1 | 2 | 2 | 11 |
| Ekeus *et al*.^35^ | 1 | 1 | 0 | 2 | 1 | 1 | 1 | 1 | 2 | 10 |
| Lindström *et al*.^36^ | 1 | 1 | 1 | 2 | 1 | 1 | 1 | 1 | 1 | 10 |
| Lindström *et al*.^37^ | 1 | 1 | 1 | 2 | 1 | 1 | 1 | 1 | 1 | 10 |
| Bilsteen *et al*.^39^ | 1 | 1 | 1 | 2 | 1 | 1 | 1 | 2 | 0 | 10 |
| Gisselmann *et al*.^38^ | 1 | 1 | 1 | 2 | 1 | 0 | 1 | 1 | 1 | 9 |
| Potijk *et al*.^40^ | 1 | 1 | 0 | 2 | 1 | 1 | 1 | 0 | 2 | 9 |
| Potijk *et al*.^41^ | 1 | 1 | 0 | 2 | 1 | 1 | 1 | 0 | 2 | 9 |
| ElHassan *et al*.^42^ | 1 | 1 | 1 | 2 | 0 | 1 | 1 | 2 | 0 | 9 |
| Lindström *et al*.^43^ | 1 | 1 | 1 | 2 | 1 | 0 | 1 | 1 | 0 | 8 |
| Ene *et al*.^44^ | 1 | 1 | 0 | 1 | 1 | 1 | 1 | 0 | 2 | 8 |
| de Laat *et al*.^45^ | 0 | 0 | 0 | 2 | 1 | 1 | 1 | 2 | 1 | 8 |
| Doyle *et al*.^46^ | 1 | 1 | 0 | 2 | 0 | 1 | 1 | 1 | 0 | 7 |
| Peacock *et al*.^47^ | 1 | 1 | 0 | 2 | 0 | 1 | 1 | 0 | 0 | 6 |
| Dall'Oglio *et al*.^48^ | 0 | 1 | 1 | 2 | 0 | 1 | 0 | 1 | 0 | 6 |
