# Supplementary material for: How do socioeconomic inequalities and preterm birth interact to modify health and education outcomes? A narrative systematic review
Source: BMJ Open. 2025 Jan 25;15(1):e084147. doi: 10.1136/bmjopen-2024-084147 (PMC11784320; doi:10.1136/bmjopen-2024-084147)
Supplement: Supplementary file 7 [file bmjopen-2024-084147.reviewer_comments.pdf]

PEER REVIEW HISTORY

BMJ Open publishes all reviews undertaken for accepted manuscripts. Reviewers are asked to complete a checklist review form and are provided with free text boxes to elaborate on their assessment. These free text comments are reproduced below.

ARTICLE DETAILS

Title (Provisional)

How do socioeconomic inequalities and preterm birth interact to modify health and education outcomes? A narrative systematic review

Authors

McHale, Philip; Fahy, Katie; Pennington , Andy; Schlüter, Daniela K; Barr, Ben; Taylor-Robinson, David

---

VERSION 1 - REVIEW

**Reviewer**	**1**
**Name**	**Boardman, James**
**Affiliation**	**The University of Edinburgh, MRC Centre for Reproductive Health**
**Date**	**02-Jul-2024**
**COI**	**I have no competing interests.**

The authors have addressed a pressing but under-researched issue in perinatal medicine: whether SES and low GA interact to shape neurodevelopmental and cognitive outcomes after premature birth. This issue is pressing because there is mounting evidence that SES is an important determinant of outcomes, but the nature of that relationship, i.e., type of SES exposure and effect size, is poorly understood. This makes optimal modelling of SES in outcome studies challenging and hampers efforts to target support. The authors have a solid track record in this area.

Using systematic review methodology, they report that the weight of current evidence based on powered studies suggests that low SES exacerbates the effect of low GA on neurodevelopment, mental health and cognition.

The review protocol was registered on PROSPERO. I agree with the authors that the minor changes to protocol as described in Appendix B are justified and do not impact the reliability of findings. Furthermore, the methdological approach has precedence (ref 16). The report conforms with PRISMA guidelines.

I have some requests and suggestions aimed at improving the manuscript.

Line 81: there is evidence that sex modifies neurodevelopment and cognition after preterm birth. Given the focus of this review, I suggest citing that literature rather than using asthma as the exemplar disease.

Line 156: Provide the possible range of scores using the Liverpool University QA Tool. It needs to be clarified what the maximum score could be based on the description in Appendix D.

In Appendix D, much more detail is needed about how scores for selection bias, response bias, measurement bias in PTB, follow-up bias and confounding were assigned. Please provide the criteria for score allocation in each category, especially those open to subjectivity, e.g., 'adequate' versus 'comprehensive' adjustment for confounding. Why did studies using area-level measures of SES necessarily score lower on quality on their scale compared with those that used individual-level measures?

I think there may be some inaccuracies in how studies have been described and, therefore, synthesised. For example, Ene et al. reported independent associations between increased deprivation and speech, language and communication concern, but "no significant association" is written in Table 3 in the "Effect of SES" column. Further, that study reported that preterm birth and neighbourhood deprivation interact additively to produce greater risk among children with both exposures. However, the manuscript states, "Lower GA was associated with higher levels of concern, while SES was not associated. No significant additive interaction was found" in lines 286-289. In the same study, language spoken in the home was modelled, but adjustment for potential confounds is scored as '0' in Appendix E. Please check the accuracy of the information for *all* studies listed in Appendix E and Table 3 and the corresponding descriptions of studies in the text.

For consideration in discussion. Did the authors find any evidence that family-level measures of SES (i.e. parental education/occupation/income) exert the greatest influence in infancy/preschool and that as children grow up, the effect of neighbourhood-level measures of SES (e.g., deprivation indices) become more important determinants? Similarly, could the level by which SES was ascertained have influenced interactions with GA? If their study elucidates these issues or other types of SES patterning, it would be useful to discuss them because it could help inform future study designs.

**Reviewer**	**2**
**Name**	**Hesson, Ashley**
**Affiliation**	**University of Michigan**
**Date**	**07-Jul-2024**
**COI**	**None**

Overall, the authors provide a thorough review on the topic of interaction between socioeconomic status and preterm birth as it relates to poor educational and health outcomes. They follow a well-described, systematic approach to their review. They detailed their adherence and deviations from relevant standards for systematic review.

My comments largely pertain to readability of the manuscript. Some terms are used with inadequate context. The discussion is also lacking in synthesis. I recommend the following edits:

Line 10- revise "How are socioeconomic inequalities modified by, or interact with, preterm birth?" to "...modified by, or how do they interact with"

Line 267- explain "consecutive survivors", this does not make sense

Line 391- if a model is referenced, it should be described in terms of its applicability to the work presented here. The discussion would benefit from additional conceptual framing, either via further incorporation of this model or invocation of another pertinent framework to synthesize the results.

Thank you for the opportunity to review this work.

**Reviewer**	**3**
**Name**	**Spiegler, Juliane**
**Affiliation**	**Universitatsklinikum Wurzburg**
**Date**	**27-Sep-2024**
**COI**	**non**

Thank you for the possibility to review this manuscript. It addresses an important topic. These data are needed to justify increased developmental mental screening in the high risk population of those born preterm with low socioeconomic status.

I am concerned about the screening process. According to the flow chart only half of the included studies have been identified by a systematic search (18 included and 10 added from other resources). This means the review process was fauly and further studies might have been missed. This is discussed in the limitation. However, can you really call this a systematic review any more?

The list with citation of excluded studies with reason from the full text screenin is missing.

There were only few abstracts screened double. Please give more detail about the "high" agreement x/y abstracts agreed/disagreed.

Perhap table 3 could give an indication of the data-source (national registry, national cohort study etc)

The quality tool needs to be explained in more detail. the citation leads to another study that used this instrument which cites as source an oral communication. Please give more details. What is the highes possible score?

I am not firm enough in data synthesis for these data. Is it really not possible to analyse the effect? Even though different SES measures are used these are always highly correlated and should lead to a similar effect.

VERSION 1 - AUTHOR RESPONSE

**Reviewer: 1**	
The authors have addressed a pressing but under-researched issue in perinatal medicine: whether SES and low GA interact to shape neurodevelopmental and cognitive outcomes after premature birth. This issue is pressing because there is mounting evidence that SES is an important determinant of outcomes, but the nature of that relationship, i.e., type of SES exposure and effect size, is poorly understood. This makes optimal modelling of SES in outcome studies challenging and hampers efforts to target support. The authors have a solid track record in this area.<br><br>Using systematic review methodology, they report that the weight of current evidence based on powered studies suggests that low SES exacerbates the effect of low GA on neurodevelopment, mental health and cognition.<br><br>The review protocol was registered on PROSPERO. I agree with the authors that the minor changes to protocol as described in Appendix B are justified and do not impact the reliability of findings. Furthermore, the methdological approach has precedence (ref 16). The report conforms with PRISMA guidelines.	Thank you for this summary
I have some requests and suggestions aimed at improving the manuscript.<br><br>Line 81: there is evidence that sex modifies neurodevelopment and cognition after preterm birth. Given the focus of this review, I suggest citing that literature rather than using asthma as the exemplar disease.<br><br>Line 156: Provide the possible range of scores	Thank you for these comments, we have amended the manuscript as follows:<br><br>Added this to the introduction:<br>*and the effect of preterm birth on neurodevelopmental outcomes,*[1]<br><br><br><br><br>Included this in the methods:

using the Liverpool University QA Tool. It needs to be clarified what the maximum score could be based on the description in Appendix D.	*Maximum score was 12.*
In Appendix D, much more detail is needed about how scores for selection bias, response bias, measurement bias in PTB, follow-up bias and confounding were assigned. Please provide the criteria for score allocation in each category, especially those open to subjectivity, e.g., 'adequate' versus 'comprehensive' adjustment for confounding. Why did studies using area-level measures of SES necessarily score lower on quality on their scale compared with those that used individual-level measures?	Appendix D has been updated to explain how the scoring was calculated.<br><br>Area-based measures scored less because it is prone to the ecological fallacy and evidence shows it is an underestimate of inequalities.[2]<br><br>Additional note; a number of studies include a number of covariates but did not include ethnicity, thus only scored 1 for confounding.
I think there may be some inaccuracies in how studies have been described and, therefore, synthesised. For example, Ene et al. reported independent associations between increased deprivation and speech, language and communication concern, but "no significant association" is written in Table 3 in the "Effect of SES" column. Further, that study reported that preterm birth and neighbourhood deprivation interact additively to produce greater risk among children with both exposures. However, the manuscript states, "Lower GA was associated with higher levels of concern, while SES was not associated. No significant additive interaction was found" in lines 286-289. In the same study, language spoken in the home was modelled, but adjustment for potential confounds is scored as '0' in Appendix E. Please check the accuracy of the information for *all* studies listed in Appendix E and Table 3 and the corresponding descriptions of studies in the text.	Thank you for this comment. We used the logistic regression in the supplementary content of Ene which adjust for gestational age and the interaction term. In this model SES was no longer significant. We have included this in our methods:<br>*For studies which included multiple models, we used results from the model which included both PTB/GA, the measure of SES and the interaction term for main effects, if this was available.*<br><br>The additive interaction was significant for SES and English as a first language but not SES and GA.<br><br>The 0 score was for no or limited adjustment, and this was agreed by both reviewers to be limited.<br><br>Results have been rechecked and no errors were identified, expanded for Doyle to increase clarity and other minor changes have been made to table 3 to increase clarity. These have had no effect on the narrative synthesis.
For consideration in discussion. Did the authors find any evidence that family-level measures of SES (i.e. parental education/occupation/income) exert the greatest influence in infancy/preschool and that as children grow up, the effect of neighbourhood-level measures of SES (e.g., deprivation indices) become more important determinants? Similarly, could the level by which SES was ascertained have influenced interactions with GA? If their study elucidates these issues or other types of SES patterning, it	We agree this would be an area of interest however this was not possible with the evidence available. We have highlighted this in our discussion:<br>*The effects of a potential interaction by age at which the outcome occurs, and the measure of SES would merit investigation.*

would be useful to discuss them because it could help inform future study designs.	
**Reviewer: 2**	
Overall, the authors provide a thorough review on the topic of interaction between socioeconomic status and preterm birth as it relates to poor educational and health outcomes. They follow a well-described, systematic approach to their review. They detailed their adherence and deviations from relevant standards for systematic review.	Thank you for the summary of our study.
My comments largely pertain to readability of the manuscript. Some terms are used with inadequate context. The discussion is also lacking in synthesis. I recommend the following edits:<br>Line 10- revise "How are socioeconomic inequalities modified by, or interact with, preterm birth?" to "...modified by, or how do they interact with"<br>Line 267- explain "consecutive survivors", this does not make sense<br><br>Line 391- if a model is referenced, it should be described in terms of its applicability to the work presented here. The discussion would benefit from additional conceptual framing, either via further incorporation of this model or invocation of another pertinent framework to synthesize the results.<br><br>Thank you for the opportunity to review this work.	This has been changed in the manuscript<br><br>We have updated this to be clearer:<br>*consecutive extremely preterm or extremely low birth weight births born during the study period who survived*<br><br>we have added an explanation to the introduction of the Diderichsen model:<br>*The theoretical basis for this research is the Diderichsen model of health inequalities.*[3] *This suggest health inequalities are generated by a set of differentials, with this study focussing on the concepts of differential susceptibility (does preterm birth make children born in low socioeconomic circumstances more susceptible to the negative outcomes) and differential disease consequence (are the negative consequences of preterm birth exacerbated by being born in low socioeconomic circumstances).*<br><br>We have clarified in the discussion what the results mean in relation to this model:<br>*The findings of this review suggest that children born preterm are more susceptible to the negative consequences of low SES for cognitive and mental health outcomes (differential susceptibility), or the consequences of being born preterm are exacerbate by low SES (differential disease consequences)*
**Reviewer: 3**	

Thank you for the possibility to review this manuscript. It addresses an important topic. These data are needed to justify increased developmental mental screening in the high risk population of those born preterm with low socioeconomic status.<br><br>I am concerned about the screening process. According to the flow chart only half of the included studies have been identified by a systematic search (18 included and 10 added from other resources). This means the review process was fauly and further studies might have been missed. This is discussed in the limitation. However, can you really call this a systematic review any more?	Thank you for this comment. Consistent with best practice for identifying relevant studies for inclusion in systematic reviews of complex social determinants of health inequalities, which have to overcome greater difficulties in identifying/locating studies because of greater variation in terms and sources compared to searches for evidence in reviews of more tightly conceptualised and defined exposures and interventions (e.g., clinical interventions), we conducted both iterative, comprehensive, targeted searches of appropriate gold standard databases and extensive supplementary searches, including rounds of forward and backward citation searching and advanced Google searches.[4–6] We also followed established practices to screening the results of searches that involved independent dual screening of approximately 15% of initial titles and abstracts followed by a calibration exercise to ensure ongoing consistency in the screening of remaining titles and abstracts by an experienced systematic reviewer. As well as potentially identifying additional relevant papers, supplementary search approaches also presented multiple, later opportunities for any relevant articles to be identified and included. Study identification approaches were informed by the Centre for Reviews and Dissemination's (CRD) guide to undertaking systematic reviews, guidance on the Conduct of Narrative Synthesis in Systematic Reviews, and the Preferred Reporting Items for Systematic Reviews and Meta-Analyses (PRISMA) and PRISMA-Equity Extension (PRISMA-E) checklists and statements.[6–10] The description of approaches for this review were checked by the CRD during the process of the submission of the review protocol to PROSPERO.<br><br>In this review, a particular focus on supplementary search methods was required because we not only had to identify papers on socioeconomic inequalities in preterm birth, but also had to identify and only include studies that examined interaction with specific relevant methods that are seldom shown in the titles, abstracts, keywords, MESH terms etc that are indexed and searched in databases. This presented a greater challenge for searching than in most systematic reviews. It is a strength

	of this review that we used sophisticated tried and tested approaches to identifying hard to locate evidence. These approaches were originally developed in reviews of theory on complex social determinants of health inequalities which face similar challenges,[11–13] before application in systematic reviews of hard to identify bodies of evidence on complex social determinants of health inequalities.[4,14,15] The studies in the PRISMA flowchart described as from "other sources" were identified during supplementary searches and screening, and not missed during initial database screening.<br><br>We have amended the limitations section to better reflect this:<br>*Some studies in our review included interaction as an additional element to the analysis. It is therefore possible that we may have missed some papers because titles and abstracts were screened for those mentioning interaction or stratification. If not mentioned, studies did not make it through to full-text screening, and therefore some valid studies may potentially have been missed. Given this, our focus on supplementary search strategies is valuable to comprehensively identifying relevant evidence. Such an approach has been applied previously to identify hard to find evidence on health inequalities.*[4,14,15]
The list with citation of excluded studies with reason from the full text screenin is missing. There were only few abstracts screened double. Please give more detail about the "high" agreement x/y abstracts agreed/disagreed.	The reasons for those excluded at full text screening is included in the PRISMA diagram. An appendix with the list of studies excluded has been added.<br>Percentage of agreement (>99%) has been added to Appendix B
Perhap table 3 could give an indication of the data-source (national registry, national cohort study etc)	We have added this information to table 2
The quality tool needs to be explained in more detail. the citation leads to another study that used this instrument which cites as source an oral communication. Please give more details. What is the highes possible score?	Appendix D has been updated to explain how the scoring was calculated.
I am not firm enough in data synthesis for these data. Is it really not possible to analyse the effect? Even though different SES measures are used these are always highly correlated and should lead to a similar effect.	Due to the lack of information in many of the studies on the interaction effect, further data synthesis (e.g. using an albatross plot) beyond a narrative synthesis was not possible.

1. Christensen R, Chau V, Synnes A, Guo T, Ufkes S, Grunau RE, et al. Preterm Sex Differences in Neurodevelopment and Brain Development from Early Life to 8 Years of Age. J Pediatr. 2025 Jan 1;276:114271.

2. Moss JL, Johnson NJ, Yu M, Altekruse SF, Cronin KA. Comparisons of individual- and area-level socioeconomic status as proxies for individual-level measures: evidence from the Mortality Disparities in American Communities study. Popul Health Metr. 2021 Jan 7;19(1):1.

3. Diderichsen F, Andersen I, Manuel C, Andersen AMN, Bach E, Baadsgaard M, et al. Health Inequality - determinants and policies. Scand J Public Health. 2012 Nov 1;40(8_suppl):12–105.

4. Pennington A, Maudsley G, Whitehead M. The impacts of profound gender discrimination on the survival of girls and women in son-preference countries - A systematic review. Health Place. 2023 Jan 1;79:102942.

5. Mays N, Pope C, Popay J. Systematically reviewing qualitative and quantitative evidence to inform management and policy-making in the health field. J Health Serv Res Policy. 2005;10:6–20.

6. CRD. Systematic reviews: CRD's guidance for undertaking reviews in health care. York: Centre for Reviews and Dissemination, University of York; 2009.

7. Popay J, Robertss H, Sowden A, Petticrew M, Arai L, Rodgers M, et al. Guidance on the Conduct of Narrative Synthesis in Systematic Reviews – A Product from the ESRC Methods Programme. Lancaster: Lancaster University; 2006.

8. Page MJ, McKenzie JE, Bossuyt PM, Boutron I, Hoffmann TC, Mulrow CD, et al. The PRISMA 2020 statement: an updated guideline for reporting systematic reviews. BMJ. 2021 Mar 29;372:n71.

9. Moher D, Liberati A, Tetzlaff J, Altman DG. Preferred reporting items for systematic reviews and meta-analyses: the PRISMA statement. BMJ. 2009 Jul 21;339:b2535.

10. Welch V, Petticrew M, Tugwell P, Moher D, O'Neill J, Waters E, et al. PRISMA-Equity 2012 Extension: Reporting Guidelines for Systematic Reviews with a Focus on Health Equity. PLOS Med. 2012 Oct 30;9(10):e1001333.

11. Lorenc T, Clayton S, Neary D, Whitehead M, Petticrew M, Thomson H, et al. Crime, fear of crime, environment, and mental health and wellbeing: Mapping review of theories and causal pathways. Infect Insecurities. 2012 Jul 1;18(4):757–65.

12. Bonell C, Jamal F, Harden A, Wells H, Parry W, Fletcher A. Systematic review of the effects of schools and school environment interventions on health: evidence mapping and synthesis. Public Health Res [Internet]. 2013 Jun 18;1(1). Available from: http://journalslibrary.nihr.ac.uk/phr/phr01010

13. Whitehead M, Pennington A, Orton L, Nayak S, Petticrew M, Sowden A, et al. How could differences in 'control over destiny' lead to socio-economic inequalities in health? A synthesis of theories and pathways in the living environment. Health Place. 2016 May 1;39:51–61.

14. Pennington A, Orton L, Nayak S, Ring A, Petticrew M, Sowden A, et al. The health impacts of women's low control in their living environment: A theory-based systematic review of

observational studies in societies with profound gender discrimination. Health Place. 2018 May 1;51:1–10.

15. Orton LC, Pennington A, Nayak S, Sowden A, Petticrew M, White M, et al. What is the evidence that differences in 'control over destiny' lead to socioeconomic inequalities in health? A theory-led systematic review of high-quality longitudinal studies on pathways in the living environment. J Epidemiol Community Health. 2019 Oct 1;73(10):929.

VERSION 2 - REVIEW

**Reviewer**	**3**
**Name**	**Spiegler, Juliane**
**Affiliation**	**Universitatsklinikum Wurzburg**
**Date**	**17-Nov-2024**
**COI**	

Thanks for this revision. My concerns have been adequately addressed
